# Responses of Grain Yield and Yield Related Parameters to Post-Heading Low-Temperature Stress in *Japonica* Rice

**DOI:** 10.3390/plants10071425

**Published:** 2021-07-12

**Authors:** Iftikhar Ali, Liang Tang, Junjie Dai, Min Kang, Aqib Mahmood, Wei Wang, Bing Liu, Leilei Liu, Weixing Cao, Yan Zhu

**Affiliations:** 1 National Engineering and Technology Center for Information Agriculture, Nanjing Agricultural University, Nanjing 210095, China; ifi.agronomist@gmail.com (I.A.); tangl@njau.edu.cn (L.T.); junjie_d@sina.cn (J.D.); 2020101189@stu.njau.edu.cn (M.K.); aqib2028@gmail.com (A.M.); 2018201094@njau.edu.cn (W.W.); bingliu@njau.edu.cn (B.L.); liulelei@njau.edu.cn (L.L.); caow@njau.edu.cn (W.C.); 2 Engineering Research Center of Smart Agriculture, Ministry of Education, Nanjing Agricultural University, Nanjing 210095, China; 3 Key Laboratory for Crop System Analysis and Decision Making, Ministry of Agriculture and Rural Affairs, Nanjing Agricultural University, Nanjing 210095, China; 4 Jiangsu Key Laboratory for Information Agriculture, Nanjing Agricultural University, Nanjing 210095, China; 5 Jiangsu Collaborative Innovation Center for Modern Crop Production, Nanjing Agricultural University, Nanjing 210095, China; 6 Department of Agronomy, University of Agriculture, Faisalabad 38000, Pakistan

**Keywords:** accumulated cold degree days, canopy temperature, flowering, grain filling, spikelet fertility, response surface model, rice yield

## Abstract

There is unprecedented increase in low-temperature stress (LTS) during post-heading stages in rice as a consequence of the recent climate changes. Quantifying the effect of LTS on yields is key to unraveling the impact of climatic changes on crop production, and therefore developing corresponding mitigation strategies. The present research was conducted to analyze and quantify the effect of post-heading LTS on rice yields as well as yield and grain filling related parameters. A two-year experiment was conducted during rice growing season of 2018 and 2019 using two *Japonica* cultivars (Huaidao 5 and Nanjing 46) with different low-temperature sensitivities, at four daily minimum/maximum temperature regimes of 21/27 °C (T1), 17/23 °C (T2), 13/19 °C (T3) and 9/15 °C (T4). These temperature treatments were performed for 3 (D1), 6 (D2) or 9 days (D3), at both flowering and grain filling stages. We found LTS for 3 days had no significant effect on grain yield, even when the daily mean temperature was as low as 12 °C. However, LTS of between 6 and 9 days at flowering but not at filling stage significantly reduced grain yield of both cultivars. Comparatively, Huaidao 5 was more cold tolerant than Nanjing 46. LTS at flowering and grain filling stages significantly reduced both maximum and mean grain filling rates. Moreover, LTS prolonged the grain filling duration of both cultivars. Additionally, there was a strong correlation between yield loss and spikelet fertility, spikelet weight at maturity, grain filling duration as well as mean and maximum grain filling rates under post-heading LTS (*p* < 0.001). Moreover, the effect of post-heading LTS on rice yield can be well quantified by integrating the canopy temperature (CT) based accumulated cold degree days (ACDD_CT_) with the response surface model. The findings of this research are useful in modeling rice productivity under LTS and for predicting rice productivity under future climates.

## 1. Introduction

Almost half of the global population consume rice [1]. *Japonica* and *Indica* are two main subspecies of Asian rice (*Oryza sativa* L.) widely cultivated throughout the world [2]. Low temperature between 12–15 °C can affect rice production [3,4], given its tropical and subtropical origin [5]. Yield losses in rice resulting from low-temperature stress (LTS) are well documented in Japan [6], Korea [7], China [8,9,10,11] and Australia [12]. Tolerance to cold differs among rice cultivars. For instance, *Japonica* rice from Korea displays greater cold tolerance than most varieties from Russia and China [13]. The cold tolerance property of rice not only depends on origin or subtype, but also on the growth stage as well as duration and intensity of LTS [14,15]. The expected higher frequency and extreme temperature patterns under recent climatic changes are poised to exacerbate the negative impacts of LTS on rice production [16]. The need to evaluate and quantify the effect of LTS on rice yield is motivated by the projected future climate changes and their impact on agricultural production.

Numerous studies have been conducted to identify cold-resistant cultivars, growth stages most sensitive to LTS and mechanisms underlying effects of LTS on rice yields. LTS causes loss of rice yields in several ways [17]. LTS during germination causes seedling death and delayed crop establishment [18], whereas during the vegetative stage, LTS reduces rice yield by reducing rice biomass [15]. LTS during jointing reduces grain yield by decreasing the number of spikelet per panicle [19]. LTS during booting stage induces male sterility and reduces the number of spikelet per panicle [3,20]. During heading and flowering stages, LTS causes poor panicle exertion, which disrupts anther dehiscence and pollination, resulting in spikelet sterility [19,21]. *Japonica* rice needs much longer grain filling time at daily mean air temperature of 21 °C, and the process may not be completed even in 75 days after flowering at temperatures under 17 °C [22]. The entire rice reproductive process from gamete formation, fertilization and grain development is sensitive to LTS [23], with the last pre-heading and the first post-heading weeks being most sensitive [24]. Extreme low temperature at the flowering and booting stages severely decreases spikelet fertility [25].

However, yield losses due to LTS should be interpreted with caution, having observed significant difference between rice under natural less stressful field conditions and those under controlled conditions with more stringent and intense stress conditions [26]. Therefore, more experiments in chambers with wide temperature variations during key growth stages are needed to effectively assess the impact of LTS on long-term food security. Even though models for quantifying the impact of cold and heat stresses on rice production have been developed [3,27,28,29,30], they rely on the cumulative cold or hot degree days below or above the optimum levels during key growth and development stages [31]. Accumulated cold degree days (ACDD) is one of the simple growth models that has been used in estimating the serious negative effects of low-temperature stress at booting on rice yield in northern Japan [32]. A modified stage-dependent model was developed where ACDD were calculated using water temperature (standing water in the paddy field) under cool climates in describing the effects of LTS at booting in *Japonica* rice [6]. However, the sensitivity of flowering and grain filling stages to LTS are less defined [15]. Even though the subroutine of the ORYZA model for cold-induced sterility during flowering is empirically derived from the ACDD, it does not compensate for cold-induced sterility in environments with wide diurnal swing temperature [33]. In general, there is need to develop models that can fully describe the above complex relationships, to improve the accuracy of estimating yield losses under extreme temperature stress [34].

The present study was designed to analyze the dynamics of yield as well as yield and grain filling related parameters in rice under moderate to extreme post-heading LTS. The impact of post-heading LTS on yield as well as yield and grain filling related parameters were also quantified by integrating the response surface model (RSM) with the canopy temperature based accumulated cold degree days (ACDD_CT_). The findings of this research are useful in estimating rice yield under variable LTS. Accordingly, the models can be used in evaluating the impact of future climate change on rice production, and therefore can inform on corresponding resilient measures.

## 2. Results

### 2.1. Effects of Post-Heading LTS on Grain Yield and Related Parameters

There was no significant difference in grain yield and yield related parameters between two experiment seasons. Yield per plant (YPP) and spikelet fertility (SF) were most influenced by low temperature level, and it was duration dependent. Post-heading LTS had no significant effect on thousand grain weight (TGW) and spikelet number per panicle (SNPP). The interaction was highly significant for most two-treatment (temperature level and duration, temperature level and cultivar, temperature level and stage, temperature duration and stage, as well as cultivar and stage) and three-treatment factors (cultivar, stage and temperature level, cultivar, stage and temperature duration, as well as stage, temperature level and duration). The interaction of four or more treatment factors was not significant. Notably, the two cultivars responded to LTS differently (Table S1). For instance, relative to Huaidao 5, the YPP and SF of Nanjing 46 decreased significantly with decreasing temperature level at the flowering stage but not grain filling stage, in a duration dependent manner. As LTS at flowering increased from T2D1 to T4D3, the YPP of Huaidao 5 and Nanjing 46 decreased by 6.39% to 38.4% and 0% to 73.6%, respectively. The duration of low temperature significantly influenced the effect of low temperature on yields. Three days duration of LTS had no significant effect on grain yield even when the daily mean temperature was as low as 12 °C (T4D1). However, moderate low temperature level (20 °C) for 9 days (T2D3) at flowering stage significantly reduced grain yields (12.5%) of Nanjing 46. Moreover, the effect of T3D3 at flowering stage on yield damage for Huaidao 5 and Nanjing 46 (15.7% and 26.9%) was comparable to that of T4D2 (12.7% and 28.1%), respectively. Similarly, yield loss was directly proportion to the duration of LTS. T4D2 at the flowering stage decreased YPP of Huaidao 5 and Nanjing 46 by 16.9% and 27.9%, respectively, whereas T4D3 caused respective 40.8% and 74.2% decrease in YPP of the two varieties (Table 1).

LTS at the flowering stage reduced yield mainly by inducing spikelet infertility, whereas the little change in YPP from LTS at the grain filling stage was due to a slight decrease in TGW. Decrease in SF was directly proportional to the duration of low temperature at the flowering stage. Notably in this regard, Nanjing 46 was more sensitive to low temperature than Huaidao 5. Particularly, the SF of Huaidao 5 and Nanjing 46 decreased by 0–41.2% and 0–73.8%, respectively, under T2D1 to T4D3 at the flowering stage. The SF of Huaidao 5 under T4D2 and T4D3 at flowering decreased from 15.5% to 41.2%, respectively, whereas that of Nanjing 46 under the same conditions deceased from 27.1% to 73.8%, respectively (Table 1).

### 2.2. Effects of Post-Heading LTS on Grain Filling

**Figure 1:**
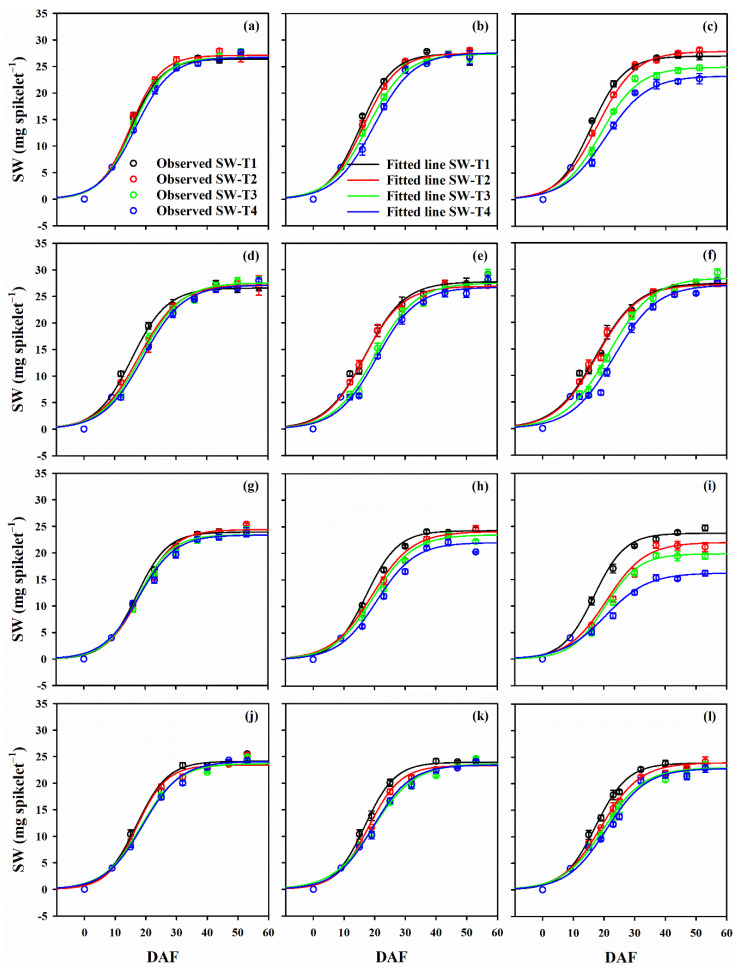
The sigmoid relationship of days after flowering (DAF) with spikelet weight (SW) in Huaidao 5 (capital letters panels) and Nanjing 46 (small letters panels) under low-temperature stress (LTS) at the flowering stage (**a**–**c**) and (**g**–**i**) grain filling stage (**d**–**f**) and (**i**–**l**) stages. The T_min_/T_max_ at T1, T2, T3 and T4 were 21/27, 17/23, 13/19 and 9/15 °C, respectively. Graph (**a**,**d**,**g**,**j**), (**b**,**e**,**h**,**k**) and (**c**,**f**,**i**,**l**) represent LTS durations of 3, 6 and 9 days, respectively. Bars on each symbol show the standard error of means. Observed values of SW_m_ were fitted against DAF using regression analysis.

For controls, grain filling started rapidly within 10–12 days after flowering (DAF) before a lag phase at 20 DAF. The final SW was achieved at 30 and 35 DAF in Huaidao and Nanjing 46, respectively. LTS at flowering delayed grain filling by 2–5 days, however, spikelet weight (SW) increased sharply thereafter (Figure 1). Moreover, LTS at the flowering stage significantly decreased the spikelet weight at maturity (SW_m_). T3 and T4 during the grain filling stage significantly impaired grain filling in Nanjing 46, and even caused retardation in Huaidao 5. However, the grain filling recovered within 5–10 days post treatment, and there was no significant decrease in SW_m_ at maturity. LTS at flowering and grain filling stages significantly increased time to 50% grain filling (t_50_) and slightly increased the steepness of curve (b) in both cultivars. LTS at these stages delayed grain filling duration from flowering to maturity (D) by 5–12 days in both cultivars. LTS decreased the maximum and mean grain filling rates (R_max_ and R_mean_) both at T3 and T4 for 6 and 9 days duration, and this decrease was more severe under LTS at flowering stage (Table 2).

### 2.3. The Correlation between Yield as Well as Yield and Grain Filling Related Parameters

**Figure 2:**
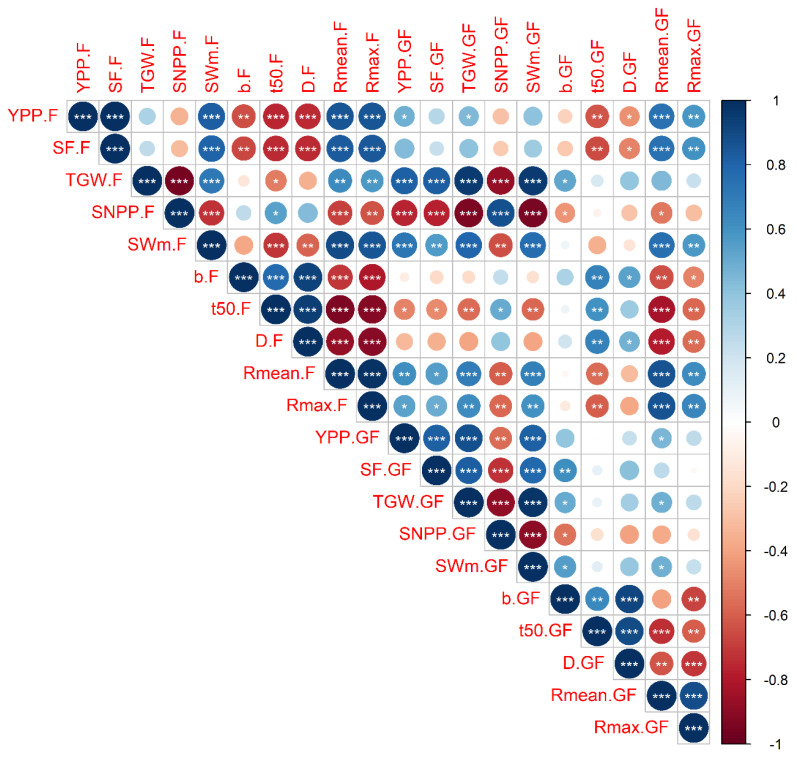
The Pearson correlation matrix between yield as well as yield and grain filling related parameters under low-temperature stress at flowering (F) and grain filling (GF) stages. (YPP) yield per plant; (SF) spikelet fertility; (TGW) thousand grain weight; (SNPP) spikelet number per panicle; (SW_m_) spikelet weight at maturity; (b) the shape or steepness of the sigmoid curve; (t_50_) days from flowering to 50% grain filling; (D) days from flowering to 95% (SW_m_) (R_mean_) grain filling rate; (R_max)_ maximum grain filling rate. *, ** and *** represent the significant correlation at *p* < 0.05, *p* < 0.01 and *p* < 0.001, respectively.

We found a positive correlation between YPP and SF (*p* < 0.001), spikelet weight at maturity (SW_m_) (*p* < 0.001) and mean grain filling rate (R_mean_) (*p* < 0.001) under LTS at both flowering and grain filling stages. In addition, under LTS at flowering stage, YPP positively correlated with the maximum grain filling rate (R_max_) but negatively correlated with the steepness of curve (b) (*p* < 0.01), days from flowering to 95% SW_m_ (D) (*p* < 0.001) and days from flowering to 50% grain filling (t_50_) (*p* < 0.001). Additionally, under LTS at the grain filling stage, YPP positively correlated with thousand grain weight (TGW) (*p* < 0.001), but negatively correlated with spikelet number per panicle (SNPP) (*p* < 0.01). The highly positive correlation of decreasing SF with SW_m_ (*p* < 0.001) under LTS at flowering stage was due to a greater number of empty spikelet and ultimately causing decreased spikelet weight (Figure 2).

### 2.4. Response Surface Model for the Association between LTS and Grain Yields as Well as Yield Related Parameters

**Figure 3:**
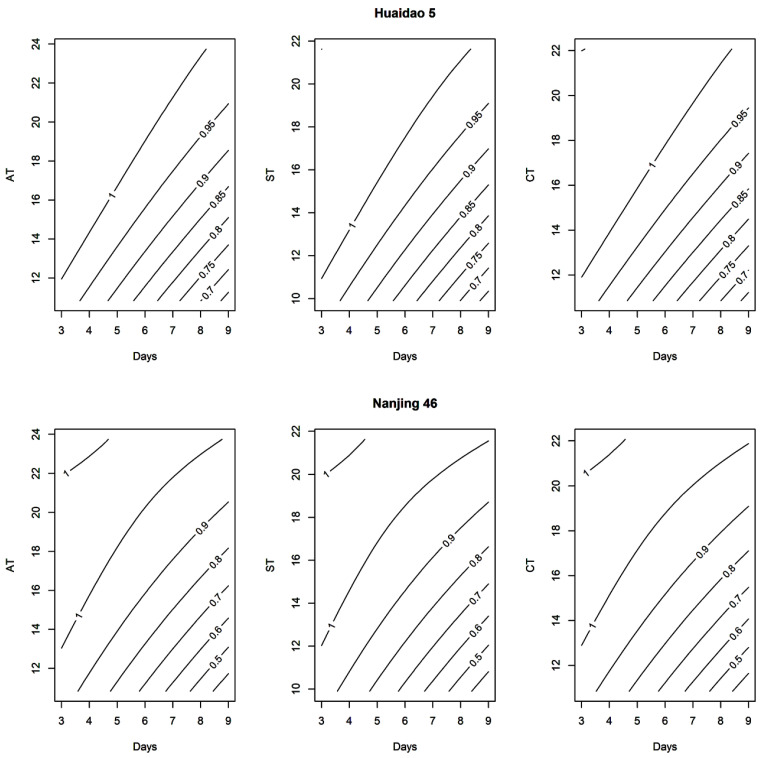
Contour plots for relative change in yield per plant (YPP) under decreasing low air temperature (AT), soil temperature (ST) and canopy temperature (CT) with increasing low-temperature stress (LTS) duration at flowering stage in Huaidao 5 and Nanjing 46.

The relative change in YPP with decreasing AT, ST and CT under increasing LTS duration perfectly fitted in the RSM (Table 3). The 10–30% decrease in YPP of Huaidao 5 was observed at 18–12 °C (AT), 16–11 °C (ST) and 17–12 °C (CT) with 9 days duration at flowering, while same decrease in YPP of Nanjing 46 was observed at 20–16 °C (AT), 18–14 °C (ST) and 18–15 °C (CT) with same LTS duration at flowering (Figure 3). Moreover, given that the effect of LTS on yield was most influenced by CT than AT and ST, we quantified the effects of LTS on yield with CT as well as on yield and grain filling related parameters. The coefficient d of RSM was highly significant (*p* < 0.001) for most of the parameters (YPP, SF, SW_m_, t_50_, R_max_, R_mean_ and D) in both varieties (Table S2), which confirmed the strong interaction of LTS duration with low temperature level. This phenomenon is depicted in contour plots for the effect of post-heading LTS on yield as well as yield and grain filling related parameters in Figures S1–S3.

### 2.5. Quantitative Effects of Post-Heading LTS on Yield and Related Parameters

**Figure 4:**
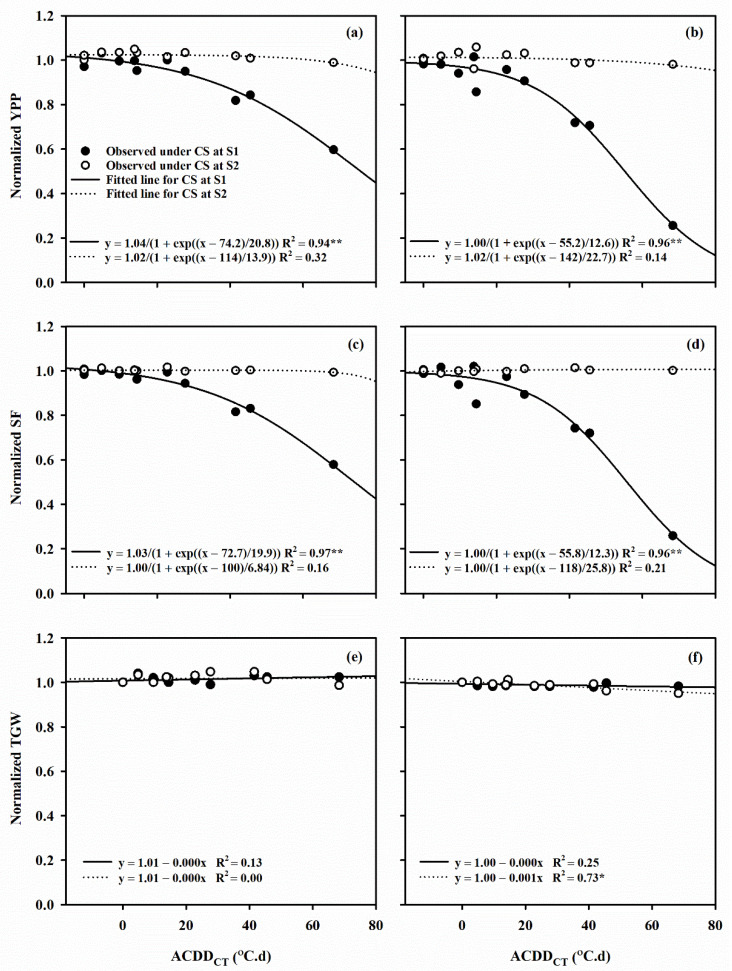
The relationship between the change in canopy temperature based accumulated cold degree days (ACDD_CT_) and the relative change in (**a**,**b**) yield per plant (YPP), (**c**,**d**) spikelet fertility (SF), (**e**,**f**) thousand grain weight (TGW) in Huaidao 5 and Nanjing 46, respectively. (S1) flowering stage; (S2) grain filling stage. Capital letters represent Huaidao 5 whereas the small letters represent Nanjing 46. ** represents *p* < 0.01; * represents *p* < 0.05).

**Figure 5:**
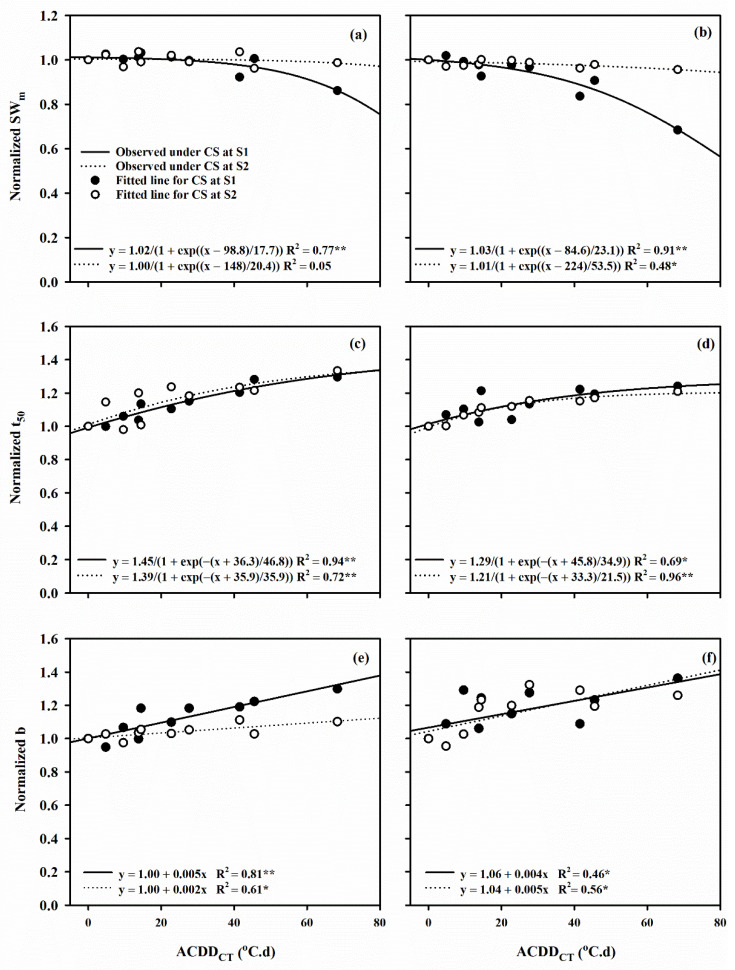
The relationship between the change in canopy temperature based accumulated cold degree days (ACDD_CT_) and the relative change in (**a**,**b**) spikelet weight at maturity (SW_m_), (**c**,**d**) days from flowering to 50% grain filling (t_50_), (**e**,**f**) shape or steepness of curve (**b**) in Huaidao 5 and Nanjing 46, respectively. (S1) Flowering stage; (S2) grain filling stage. Capital letters represent Huaidao 5 whereas the small letters represent Nanjing 46. ** represents *p* < 0.01; * represents *p* < 0.05).

**Figure 6:**
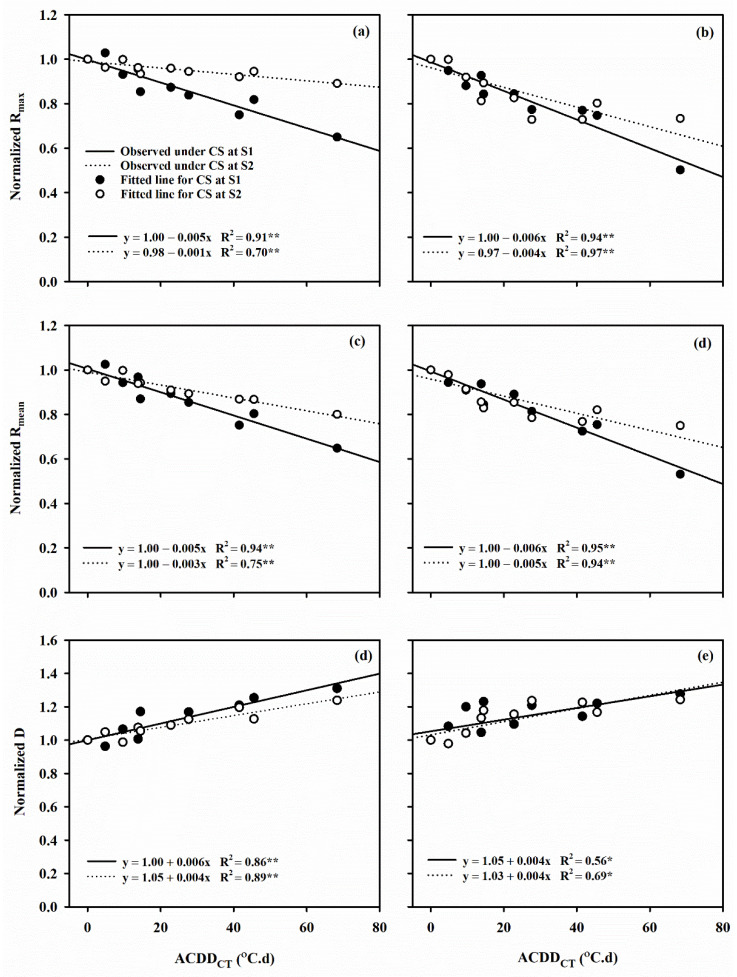
The relationship between the change in canopy temperature based accumulated cold degree days (ACDD_CT_) and the relative change in (**a**,**b**) maximum filling rate (R_max_), (**c**,**d**) mean filling rate (R_mean_) and (**e**,**f**) total days from flowering to 95% SW_m_ (D) in Huaidao 5 and Nanjing 46, respectively. (S1) Flowering stage; (S2) grain filling stage. Capital letters represent Huaidao 5 whereas the small letters represent Nanjing 46. ** represents *p* < 0.01; * represents *p* < 0.05).

The relationship between normalized YPP, SF, SW_m_ and t_50_ and ACDD_CT_ perfectly fitted into the sigmoid function: y = a/1 − exp ((x − x_0_)/b). Under LTS at flowering stage, the 50% reduction in YPP was caused by an ACDD_CT_ of 74.2 and 55.2 °C.d, in SF by 72.7 and 55.8 °C, and in SW_m_ by 98.8 and 84.6 °C.d in Huaidao 5 and Nanjing 46, respectively (Figure 4 and Figure 5). Normalized b, R_mean_, R_max_ and D with ACDD_CT_ under post-heading LTS displayed a linear relationship (y = a + bx) for both cultivars. A 1 °C.d increase in ACDD_CT_ at flowering decreased the R_mean_ of Huaidao 5 and Nanjing 46 by 0.5% and 0.3%, respectively, where similar conditions at grain filling stages resulted in a 0.6% and 0.5% increase in the R_mean_ of Huaidao 5 and Nanjing 46, respectively. On the other hand, a 1 °C.d increase in ACDD_CT_ at flowering and grain filling stages resulted in 0.5% and 0.1% decrease in R_max_ of Huaidao 5 and 0.6% and 0.4% decrease in R_max_ of Nanjing 46, respectively. Additionally, a 1 °C.d ACDD_CT_ increase at flowering stage resulted in a linear increase of 0.6% and 0.4% in D of Huaidao 5 and Nanjing 46, respectively. At the grain filling stage, a 1 °C.d ACDD_CT_ increased the D by 0.4% for both Huaidao 5 and Nanjing 46 (Figure 6).

## 3. Discussion

### 3.1. Effect of Post-Heading LTS on Yields and Related Parameters in Rice

The limiting effect of prolonged low temperature on yield has been widely reported [21,23,25,35]. Herein, we found short LTS of low temperature between 12 and 20 °C for 3 days had no significant effect on yields of both rice varieties, in contrast, the lethal effects of short-term post-heading heat stress on yield has been reported in *Japonica* rice [30,36]. However, longer LTS of 6 and 9 days at the flowering stage significantly decreased the SF or YPP of both rice varieties. We found T3D3 with less ACDD_CT_ caused greater yield loss than T4D2 with more ACDD_CT_, underling the significant negative effect of low temperature duration on yields as previously reported by [23,35]. Additionally, we found LTS at the flowering stage resulted in greater loss than at the grain filling stage. LTS at flowering mainly induced spikelet infertility, consistent with previous findings [21,24]. Flowering is one of the most critical stages in rice production. LTS affects critical events such as anthesis, anther dehiscence, pollination and fertilization [23]. Anthesis is highly sensitive to LTS, which explains high spikelet infertility under prolonged low-temperature stress. Comparatively, Nanjing 46 with longer flowering period was more sensitive to LTS at the flowering stage than Huaidao 5 with shorter flowering period. Fast germination of pollen is the cold tolerance characteristics in cultivars with shorter flowering period [37]. Varieties with short to medium grain length, typically recommended for cold regions, were found to be more tolerant to low temperatures but more sensitive to high temperatures, in comparison to cultivars with long grain length [31]. High anther dehiscence increases the rate of pollination at the flowering stage in cold tolerant genotypes, thus reduces spikelet sterility [38].

The flowering stage of *Japonica* cultivars usually lasts approximately 9 days [36]. Therefore, LTS after flowering or during grain filling only moderately influence rice yield. However, the effect of LTS at the grain filling stage cannot be ignored because low daily temperature substantially slows growth. Low temperatures of 21 °C prolonged the grain filling stage of *Japonica* rice, and in some instances, the process could not complete in 75 days at daily mean temperature of 17 °C [22]. Low temperature results in thermal retardation or permanently impairs critical growth and developmental processes, thus reducing yields [19]. In this study, we found 12–22 °C temperatures only slightly reduced final grain weight of Nanjing 46 and Huaidao 5 at maturity, consistent with previous finding [39]. LTS at the flowering stage resulted in significantly low SW because it disrupted filling of spikelet. In fact, SF positively correlated with SW (*p* < 0.001). LTS at both flowering and grain filling stages substantially decreased the mean and maximum grain filling rates (R_mean_ and R_max_) in both Nanjing 46 and Huaidao 5, attributed to thermal retardation [19]. In the present study, we found LTS for 3–9 days significantly slowed down or stopped altogether grain filling process. However, the process resumed within 5–10 days in ambient growth conditions.

### 3.2. Quantification of Post-Heading LTS Effects on Rice Yield

Rice yields are most affected by length of low temperature period [39]. Quantification of crop yield potential under changing climate condition typically rely on crop models, which are lacking in most cases due to a lack of field data [33]. Temperature sensitivity model may be key in accurately predicting the phenological responses to climate change [40]. At the same time, the impact of extreme temperature duration on yield needs to be factored in the model [41]. In addition, it is difficult to predict sterility because canopy temperature may differ from air temperature due to transpiration cooling effect [42]. The temperature in the panicles would be more significant than air temperature because the panicle is more sensitive to temperature than the other plant organs [30,32]. Herein, air temperature, canopy surface temperature and soil temperature were measured daily. We found the canopy surface temperature was more comparable to the temperature of panicles during post-heading stages. In addition, diurnal variation of canopy surface temperature was lower than that of air temperature in phytotron chambers with T1 and T2, but the difference was smaller at T3 and T4. Moreover, the greater difference between canopy and air temperature is mainly observed during midday at full sunshine [43]. We found canopy surface temperature can better quantify the effect of LTS on yield as well as yield and grain filling related parameters than soil and air temperatures.

In this study, by integrating the RSM with the canopy temperature based accumulated cold degree days (ACCD_CT_), we perfectly described (R^2^ > 0.90) the relative change in YPP, SF, SW_m_, t_50_, R_mean_ and R_max_ under varied daily mean CT and LTS duration for both cultivars, which considered the interaction between low temperature and duration. The logistic regression function described the exponential relationship of the relative change in SF, YPP, SW_m_ and t_50_ to ACDD_CT_ (R^2^ > 0.90) as previously reported [6,44]. Even though the ORYZA model for cold-induced sterility during flowering is also empirically derived from ACDDCT, it cannot quantify the cold-induced sterility in environments with large variation in diurnal temperature [33]. Notably in this study, we observed that even though T4D1 has greater ACCD_CT_ (23 °C.d) than T2D3 (15 °C.d), T2D3 caused greater impact on SF and YPP than T4D1, this observation can be well estimated by integrating the RSM with the ACCD, but still needs to be tested in rice growth models with more independent dataset from wider environments in the near future.

## 4. Materials and Methods

### 4.1. Crop Husbandry and Experimental Design

Controlled-environment sunlit phytotron experiments were conducted in Rugao (120.33° E, 32.23° N), Jiangsu Province, China, between 2018 and 2019 using two *Japonica* rice cultivars (Huaidao-5 with high resistance to cold stress and Nanjing 46 with low resistance to cold stress). Seedlings at three-leaf stage were transplanted into plastic pots (diameter 28 cm and height 25 cm) filled with 15 kg soil. The planting density was 3 hills per pot (2 seedlings per hill). The pots were grown under ambient weather conditions before LTS treatment. Basal fertilizer at the rate of 1.5 g N, 1.5 g P_2_O_5_ and 2 g K_2_O per pot was applied before transplantation. Supplemental N was top-dressed at mid-tillering and jointing stages at the rate of 0.3 g N and 1.2 g N per pot, respectively. The pots were kept flooded until one week before harvesting. Watering at the late active tillering stage was stopped to ensure efficient tillering. Weeds were removed manually, whereas pest and diseases were controlled using pesticides. LTS was designed at four temperature levels (T_min_/T_max_: 21/27 °C, 17/23 °C, 13/19 °C, and 9/15 °C) and three temperature durations (3, 6 and 9 days), at flowering and grain filling stages. The experiments were performed in four separate phytotrons. The post-heading LTS treatments are summarized in Table 4.

**Figure 7:**
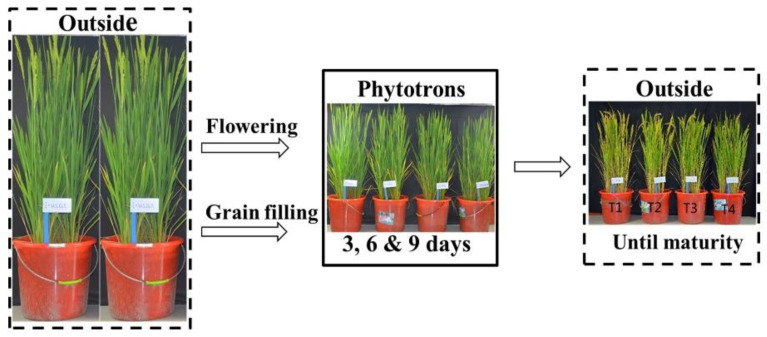
Pictorial view of the experiment design. T1, T2, T3 and T4 are temperature levels. The T_min_/T_max_ of T1, T2, T3 and T4 were 21/27, 17/23, 13/19 and 9/15 °C, respectively.

Pots with homogenous tiller number (primary + secondary) were transferred into phytotrons (L × W × H: 3.4 m × 3.2 m × 2.8 m) when 50% of panicles of each pot started flowering (flowering stage). Another set of pots was transferred into the same phytotrons 12 days after start of flowering (grain filling stage) (Figure 7). Each treatment was performed in triplicate and the treatments were completely randomized. At the end of respective treatment durations, the pots were labeled, removed from chambers and maintained under ambient conditions until maturity.

### 4.2. Ambient and Phytotron Environment

**Figure 8:**
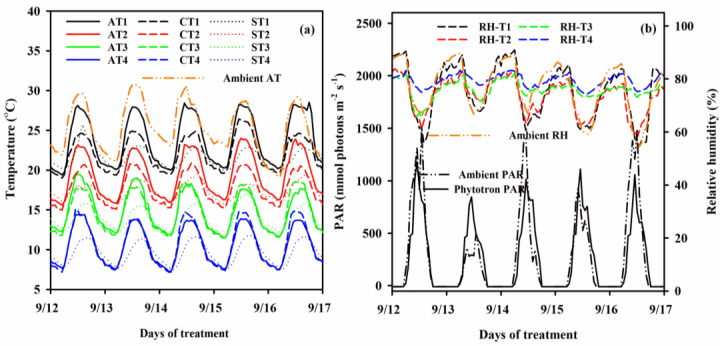
The (**a**) diurnal air (solid lines), canopy (broken lines) and soil temperatures (dotted lines) and (**b**) relative humidity (RH) (broken lines) and incident photosynthetically active radiations (PAR) (solid line) during low temperature treatment episodes in the phytotrons. Double dotted lines represent the ambient AT, PAR and RH of the same days.

Air temperature (AT) (°C), soil temperature (ST) (°C) and relative humidity (RH) (%) in the phytotrons were monitored at 10 min interval using a VP-4 sensor from METER Group, Inc. (Washington, DC, USA). The canopy surface temperature (CT, °C) and photosynthetically active radiation (PAR, μmol photons m^−2^ s^−1^) were also measured at 10 min interval using an SI-111 infrared radiometer, Apogee Instruments Inc (Logan, UT, USA) and QSO-S PAR Photon Flux sensor (Washington, DC, USA), respectively. The sensors were installed as previously described [36,45]. Diurnal AT, ST, CT, RH and PAR changes under the four temperature levels are shown in Figure 1. The CT and ST were lower than ATs. However, in T3 and T4 chambers with 6 and 9 days durations, the CT was slightly higher than AT. At T1, T2, T3 and T4, the daily average ATs were 23.8, 19.2, 14.8 and 11.2 °C, respectively. The STs were 21.6, 17.4, 14.1 and 9.9 °C, respectively, whereas the CTs were 22.2, 17.5, 14.1 and 11.1 °C, respectively (Figure 8a). The daily average PAR in chambers were about 700 μmol photons m^−2^ s^−1^ and 400 μmol photons m^−2^ s^−1^ on sunny and cloudy days, respectively. The daytime RH was about 75% and 65% at T3/T4 and T1/T2, respectively (Figure 8b).

### 4.3. Yield as Well as Yield and Grain FillingRelated Parameters

Panicles were collected every 7 days till maturity and were placed in the oven at 80 °C to achieve a constant dry weight. The panicles were divided into three parts (upper, middle and lower), with 100 spikelets picked randomly from each part, weighed and averaged for spikelet weight (SW, mg spikelet^−1^). Yield per plant (YPP; g plant^−1^) and yield components as spikelet fertility (SF%), spikelet number per panicle (SNPP) and thousand-grain weight (TGW; g) were calculated after harvesting at physiological maturity (3 pots per treatment), as previously described [30].

The dynamics of SW against days after flowering (DAF) was described using Equation (1):

(1) $$y=\frac{a}{1+e^{\left(-\frac{x-x_{0}}{b}\right)}}$$

where y is the graph of SW against DAF (x), a is the estimated SW at maturity (SW_m_; mg spikelet^−1^), x_0_ is the estimated t_50_, the time in days when achieving 50% weight of SW_m_, b is the equation coefficient which determines the shape or steepness of curve [30,46].The maximum grain filling rate (R_max_; mg spike^−1^ d^−1^), the mean grain filling rate (R_mean_; mg spike^−1^ d^−1^) and the total days from flowering to 95% SW_m_ (D; d) under each treatment were calculated based on estimates derived from Equation (1), as described in Equations (2) and (3):

(2) $$R_{\max}=\frac{a}{4b}$$

(3) $$D=-\;\ln\left(0.05\right)\times \;b\;+{\;x}_{0}$$

(4) $$R_{\mathrm{mean}}=\frac{a}{D}$$

### 4.4. Response Surface Model

Relative grain yield as well as yield and grain filling related parameters were calculated by dividing the treatment value with respective control value (T1 treatment). The relative changes in grain yield as well as yield and grain filling related parameters were fitted against decreasing low temperature level and increasing low temperature duration using RSM [47] as Equation (5):

(5) $$y\;=\;a\;+{\;\mathrm{bx}}_{1}\;+{\;\mathrm{cx}}_{2}\;+{\;\mathrm{dx}}_{1}x_{2}\;+{\;\mathrm{ex}}_{1}^{2}\;+{\;\mathrm{fx}}_{2}^{2}$$

where y is the dependent variable (relative yield, as well as yield related and grain filling related parameters), x_1_ = LTS duration (3, 6 and 9 days) in each treatment and x_2_ = daily average air (AT), or soil (ST) or canopy (CT) temperatures (independent variables), while a, b, c, d, e and f are the coefficients of RSM which were obtained after fitting the observed values of y, x_1_ and x_2_ on the above Equation (5).

### 4.5. Quantification of the Effect of Post-Heading LTS on Yields and Related Parameters

The effect of post-heading LTS on yield as well as yield and grain filling related parameters were derived using CT-based accumulated cold degree days (ACDD_CT_) as previously described [48]:

(6) $${\mathrm{ACDD}}_{\mathrm{CT}}\;=\;\overset{m}{\underset{\;i=1}{\sum }}{\mathrm{CDD}}_{i}$$

(7) $${\mathrm{CDD}}_{i}\;=\;\frac{1}{24}\overset{24}{\underset{t=1}{\sum }}{\mathrm{CD}}_{t\;}$$

(8) $${\mathrm{CD}}_{t}\;=\;\{\begin{array}{l} 0\;\;\;\;\;{\mathrm{CT}}_{t}>{\mathrm{CT}}_{h}\\ {\mathrm{CT}}_{t}-{\mathrm{CT}}_{h}\;\;{\mathrm{CT}}_{t}\leq {\mathrm{CT}}_{h}\; \end{array}$$

where CDD_i_ (°C.d) is the average hourly cold degree day at ith day under LTS treatment, CD_t_ (°C.d) is the hourly cold degree day at t hour of a day, and CT_t_ (°C) is hourly canopy temperature at t hour of a day. CT_h_ is the daily average lowest canopy temperature which reduces grain yield by 10% [15]. In this research, CT_h_ was estimated by analyzing the yield response to decreasing canopy low temperature levels and increasing low temperature duration on RSM in equation (5) and was set as 17.9 °C.

### 4.6. Statistical Analysis

Two years of experimental data were analyzed by one-way ANOVA using SigmaPlot Version 14.0 (Systat Software, San Jose, CA, USA) to estimate the effects of low temperature treatment on grain yield and yield components. The significant differences between treatments were identified at the 0.05 and 0.001 probability level (*p*) by Tukey’s test. The dynamics of SW against days after flowering (DAF) were fitted on 3 parameters sigmoid function using non-linear regression analysis in SigmaPlot Version 14.0 (Systat Software, San Jose, CA, USA). Furthermore, the association between yield or yield related parameters and grain filling related parameters was assessed based on Pearson correlation analysis using the ‘corrplot’ package in R software V. 4.0.8. Response surface analysis was conducted to assess the effects of low temperature level and duration on yield as well as yield and grain filling related parameters using ‘rsm’ package in R software, V. 4.0.8. The effects of LTS on yield as well as yield and grain filling related parameters were demonstrated using line and contour graphs.

## 5. Conclusions

In this study, we found that low temperature at the flowering and grain filling stages reduced rice yield. Huaidao 5 is an early-maturing cultivar that is more tolerant to post-heading LTS, relative to late-maturing Nanjing 46. Yields of Huaidao 5 and Nanjing 46 are both significantly influenced when canopy temperatures are lower than 17.1 and 18.6 °C, respectively. Short LTS with 3 days has no significant impact on rice yields, even if the temperature was as low as 12 °C. Contrarily, LTS of 6 to 9 days at the flowering stage severely decreased YPP, SF, SW_m_, R_max_ and R_mean_ but increased t_50_ and D. In addition, post-heading LTS has no effect on TGW and SNPP. However, LTS at the grain filling stage significantly prolonged the grain filling period but decreased grain filling rates. By incorporating these relationships, we developed a RSM with accumulated cold degree days based on canopy temperature. The model can effectively quantify the effect of post-heading LTS on rice yield as well as yield and grain filling related parameters. By facilitating modeling of yields under LTS, our model can be used in evaluating the impact of future climate change on rice productivity.

## Figures and Tables

**Table 1:** Effects of low-temperature stress at flowering and grain filling stages on yield and related parameters over the two-year experiment period.

Cultivar	LTS	Flowering Stage				Grain Filling Stage			
		SF (%)	TGW (g)	YPP (g plant^−1^)	SNPP	SF (%)	TGW (g)	YPP (g plant^−1^)	SNPP
Huaidao 5	Control	94.6 a	29.8	17.2 a	122.1	96.5	29.5	17.7	124.9
	T2D1	96.2a	31.0	18.3 a	123.0	97.3	30.5	18.7	126.0
	T2D2	94.6 a	30.4	17.7 a	123.1	96.2	29.5	18.0	126.8
	T2D3	92.5 a	29.8	16.9 a	123.1	96.1	30.1	18.3	126.9
	T3D1	96.5 a	30.3	17.7 a	120.9	96.3	30.2	18.2	125.6
	T3D2	90.7 a	29.5	16.9 a	125.8	95.9	30.9	17.9	121.3
	T3D3	78.4 b	30.7	14.5 c	120.6	96.2	30.9	18.7	126.2
	T4D1	95.6 a	30.1	17.8 a	123.4	97.7	30.4	19.2	129.2
	T4D2	79.9 b	30.5	15.0 bc	122.5	96.4	29.9	18.4	127.4
	T4D3	55.6 c	30.5	10.6 d	125.1	95.5	29.1	17.2	123.9
Statistical Significance	Year (Y)	ns	ns	ns	ns	ns	ns	ns	ns
	Temp (T)	**	ns	**	ns	ns	ns	ns	ns
	Duration (D)	**	ns	**	ns	ns	ns	ns	ns
	Y*T	ns	ns	ns	ns	ns	ns	ns	ns
	Y*D	ns	ns	ns	ns	ns	ns	ns	ns
	T*D	**	ns	**	ns	ns	ns	ns	ns
	Y*T*D	ns	ns	ns	ns	ns	ns	ns	ns
Nanjing 46	Control	91.4 a	27.3	16.7 a	134	94.6	26.3	16.8	134.9
	T2D1	94.1 a	26.9	16.7 a	131.8	93.4	26.4	17.0	138.0
	T2D2	86.8 abc	26.8	16.0 ab	137.9	94.4	26.1	17.3	140.7
	T2D3	78.8 cd	27.2	14.6 c	136.1	95	26.6	17.7	139.9
	T3D1	94.4 a	26.9	17.3 a	135.9	94.1	26	16.1	131.6
	T3D2	82.7 bc	26.8	15.4 ab	139.2	95.3	26	17.2	138.9
	T3D3	68.7 de	26.7	12.2 c	133.8	95.7	26.1	16.5	132.3
	T4D1	90.1 ab	26.8	16.3 ab	135.3	94.1	25.9	17.1	140.4
	T4D2	66.6 e	27.2	12.0 c	132.0	94.7	25.3	16.5	138.0
	T4D3	23.9 f	26.8	04.4 d	137.0	94.6	25.0	16.4	138.5
Statistical	Y	ns	ns	ns	ns	ns	ns	ns	ns
Significance	T	**	ns	**	ns	ns	*	ns	ns
	D	**	ns	**	ns	ns	ns	ns	ns
	Y*T	ns	ns	ns	ns	ns	ns	ns	ns
	Y*D	ns	ns	ns	ns	ns	ns	ns	ns
	T*D	**	ns	**	ns	ns	ns	ns	ns
	Y*T*D	ns	ns	ns	ns	ns	ns	ns	ns

Note: The data represents mean values of products or treatments followed by different letters showing significant differences between different treatments. The mean values with similar letters or without letters are statistically non-significant. (SF) spikelet fertility; (TGW) thousand grain weight; (SNPP) spikelet number per panicle; (YPP) yield per plant. T1, T2, T3 and T4 are low temperature levels. The T_min_/T_max_ for T1, T2, T3 and T4 were 21/27, 17/23, 13/19 and 9/15 °C, respectively. D1, D2 and D3 represent LTS duration of 3, 6 and 9 days, respectively. T1D2 was the control group. * represents *p* < 0.05; ** represents *p* < 0.001, while ns represents *p* > 0.05.

**Table 2:** The effect of post-heading low-temperature stress (LTS) on grain filling related parameters.

Cultivar	Stage	Treatment	SW_m_ (mg spike^−1^)	B	t_50_ (d)	*p* Value	R^2^	D (d)	R_mean_ (mg spike^−1^ d^−1^)	R_max_ (mg spike^−1^ d^−1^)
Huaidao 5	Flowering	Control	27.4	5.2	15.4	<0.0001	0.981	31.1	0.88	1.32
		T2D1	27.1	5.0	15.0	<0.0001	0.976	30.0	0.90	1.36
		T2D2	27.5	5.6	16.3	<0.0001	0.983	33.1	0.83	1.23
		T2D3	27.9	6.2	17.8	<0.0001	0.987	36.4	0.77	1.13
		T3D1	26.7	5.3	15.6	<0.0001	0.981	31.3	0.85	1.26
		T3D2	27.4	6.2	17.7	<0.0001	0.986	36.4	0.75	1.10
		T3D3	24.9	6.3	18.8	<0.0001	0.973	37.6	0.66	0.99
		T4D1	26.7	5.8	16.6	<0.0001	0.980	33.9	0.79	1.15
		T4D2	27.6	6.4	19.7	<0.0001	0.975	39.0	0.71	1.08
		T4D3	23.3	6.8	20.3	<0.0001	0.951	40.8	0.57	0.86
	Grain Filling	Control	27.8	6.4	17.0	<0.0001	0.957	36.3	0.77	1.09
		T2D1	27.2	6.5	17.9	<0.0001	0.971	37.4	0.73	1.05
		T2D2	26.9	6.2	16.7	<0.0001	0.974	35.2	0.76	1.08
		T2D3	27.2	6.7	17.6	<0.0001	0.965	37.7	0.72	1.01
		T3D1	27.6	6.6	18.7	<0.0001	0.969	38.4	0.72	1.05
		T3D2	27.5	6.7	20.1	<0.0001	0.970	40.2	0.68	1.03
		T3D3	28.4	7.1	21.5	<0.0001	0.976	42.7	0.67	1.00
		T4D1	27.1	6.5	19.3	<0.0001	0.986	38.9	0.70	1.04
		T4D2	26.7	6.5	20.7	<0.0001	0.972	40.2	0.66	1.03
		T4D3	27.1	7.0	23.2	<0.0001	0.969	44.2	0.61	0.97
Nanjing 46	Flowering	Control	24.3	5.2	17.3	<0.0001	0.983	32.9	0.74	1.17
		T2D1	24.4	5.5	18.4	<0.0001	0.979	35.0	0.70	1.11
		T2D2	24.1	6.6	19.1	<0.0001	0.965	38.8	0.62	0.91
		T2D3	21.9	6.3	20.5	<0.0001	0.936	39.8	0.55	0.87
		T3D1	23.4	5.4	17.6	<0.0001	0.977	33.8	0.69	1.08
		T3D2	23.5	6.5	19.6	<0.0001	0.971	39.1	0.60	0.90
		T3D3	19.8	5.5	20.3	<0.0001	0.939	37.0	0.54	0.90
		T4D1	23.3	5.9	17.9	<0.0001	0.975	35.4	0.66	0.99
		T4D2	22.0	6.3	20.7	<0.0001	0.971	39.5	0.56	0.87
		T4D3	16.2	6.9	20.8	<0.0001	0.951	41.3	0.39	0.59
	Grain Filling	Control	23.9	5.0	16.8	<0.0001	0.978	31.6	0.76	1.20
		T2D1	23.4	4.9	17.0	<0.0001	0.976	31.6	0.74	1.19
		T2D2	23.3	5.3	17.9	<0.0001	0.981	33.7	0.69	1.10
		T2D3	23.9	6.3	19.2	<0.0001	0.964	38.1	0.63	0.95
		T3D1	23.7	6.1	18.4	<0.0001	0.983	36.6	0.65	0.97
		T3D2	23.7	6.8	19.4	<0.0001	0.985	39.9	0.59	0.87
		T3D3	23.0	6.6	19.8	<0.0001	0.964	39.6	0.58	0.87
		T4D1	24.1	6.1	18.9	<0.0001	0.981	37.3	0.65	0.99
		T4D2	23.4	6.1	19.6	<0.0001	0.978	37.7	0.62	0.96
		T4D3	22.8	6.5	20.8	<0.0001	0.961	40.2	0.57	0.88

Note: T1, T2, T3 and T4 are temperature levels. The T_min_/T_max_ of T1, T2, T3 and T4 were 21/27, 17/23, 13/19 and 9/15 °C, respectively. D1, D2 and D3 represent low-temperature stress duration of 3, 6 and 9 days, respectively. T1D2 was the control group. SW_m_: the spikelet weight at maturity; b: the shape or steepness of the sigmoid curve; t_50_: days from flowering to 50% grain filling; (D) total days from flowering to 95% SW_m_; R_mean_: the mean grain filling rate; (R_max_) the maximum grain filling rate.

**Table 3:** Parameters of response surface model (RSM) for the relationship of low air temperature (AT), soil temperature (ST) and canopy temperature (CT) with grain yield of Huaidao 5 and Nanjing 46 at flowering stage under varied low-temperature stress.

Cultivar		Air Temperature (AT)		Soil Temperature (ST)		Canopy Temperature (CT)	
	Model Parameters	Estimate	Significance	Estimate	Significance	Estimate	Significance
Huaidao 5	a	0.8599	0.0038 **	0.8775	0.0036 **	0.7767	0.0146 *
	b	−0.0772	0.0491 *	−0.0787	0.0501	−0.0845	0.0499 *
	c	0.0363	0.105	0.0377	0.1201	0.0493	0.0935
	d	0.0044	0.0019 **	0.0049	0.0021 **	0.0051	0.0028 **
	e	−0.0024	0.3439	−0.0024	0.3534	−0.0024	0.3757
	f	−0.0014	0.0395 *	−0.0016	0.0436 *	−0.0019	0.0388 *
	**R^2^**	**0.9601**		**0.9584**		**0.954**	
Nanjing 46	a	0.8135	0.0573	0.8395	0.0364*	0.6942	0.1168
	b	−0.1383	0.0542	−0.1414	0.0372*	−0.1531	0.0365 *
	c	0.0534	0.1797	0.0552	0.1586	0.0725	0.1273
	d	0.0085	0.0015 **	0.0095	0.0009 ***	0.0099	0.0012 **
	e	−0.0047	0.3106	−0.0047	0.2707	−0.0047	0.2943
	f	−0.0023	0.0597	−0.0026	0.045 *	−0.0031	0.0426 *
	**R^2^**	**0.9183**		**0.9319**		**0.9241**	

*** represents *p* < 0.001; ** represents *p* < 0.01; * represents *p* < 0.05.

**Table 4:** Summary of the post-heading low-temperature stress treatments.

Cultivar	Stage	Temperature (T_min_/T_max_) (°C)	Duration (days)	Start of Treatment	End of Treatment
Huaidao 5	Flowering	T1 (21/27), T2 (17/23), T3 (13/19) and T4 (09/15)	D1 (3), D2 (6) and D3 (9)	08/23 in 2018 08/26 in 2019	09/01 in 2018 09/04 in 2019
	Grain Filling			09/02 in 2018 09/08 in 2019	09/11 in 2018 09/17 in 2019
Nanjing 46	Flowering			09/08 in 2018 09/12 in 2019	09/17 in 2018 09/21 in 2019
	Grain Filling			09/21 in 2018 09/23 in 2019	09/30 in 2018 10/02 in 2019

Note: T is the temperature level; D is the stress duration; T_min_ is the daily minimum temperature; T_max_: daily maximum temperature.

## Data Availability

All data generated or analyzed during this study are included in this article.

## Supplementary Materials

The following are available online at https://www.mdpi.com/article/10.3390/plants10071425/s1, Figure S1: Contour plots for relative change in spikelet weight at maturity (SW_m_), days from flowering to 50% grain filling (t_50_) and shape or steepness of sigmoid curve (b) under varied low-temperature stresses at flowering (A/a) and grain filling (B/b) stages in Huaidao 5 (A/B) and Nanjing 46 (a/b), Figure S2: Contour plots for relative changes in maximum grain filling rate (R_max_), mean grain filling rate (R_mean_) and the total days from flowering to 95% SW_m_ (D) under varied low-temperature stresses at flowering (A/a) and grain filling (B/b) stages in Huaidao 5 (A/B) and Nanjing 46 (a/b), Figure S3: Contour plots for relative changes in yield per plant (YPP), spikelet fertility (SF) and thousand grain weight (TGW) under varied low-temperature stresses at flowering (A/a) and grain filling (B/b) stages in Huaidao 5 (A/B) and Nanjing 46 (a/b), Table S1: Variance analysis of grain yield and related parameters under post-heading low-temperature stress during two-year experiments in 2018-2019, Table S2: Parameters of the RSM fitted on yield as well as yield and grain filling related parameters under varied low-temperature stresses
