# Supplementary material for: Responses of Grain Yield and Yield Related Parameters to Post-Heading Low-Temperature Stress in Japonica Rice
Source: Plants (Basel). 2021 Jul 12;10(7):1425. doi: 10.3390/plants10071425 (PMC8309334; doi:10.3390/plants10071425)
Supplement: Supplementary file 1 [file plants-10-01425-s001.zip › plants-1269491-supplementary.pdf]

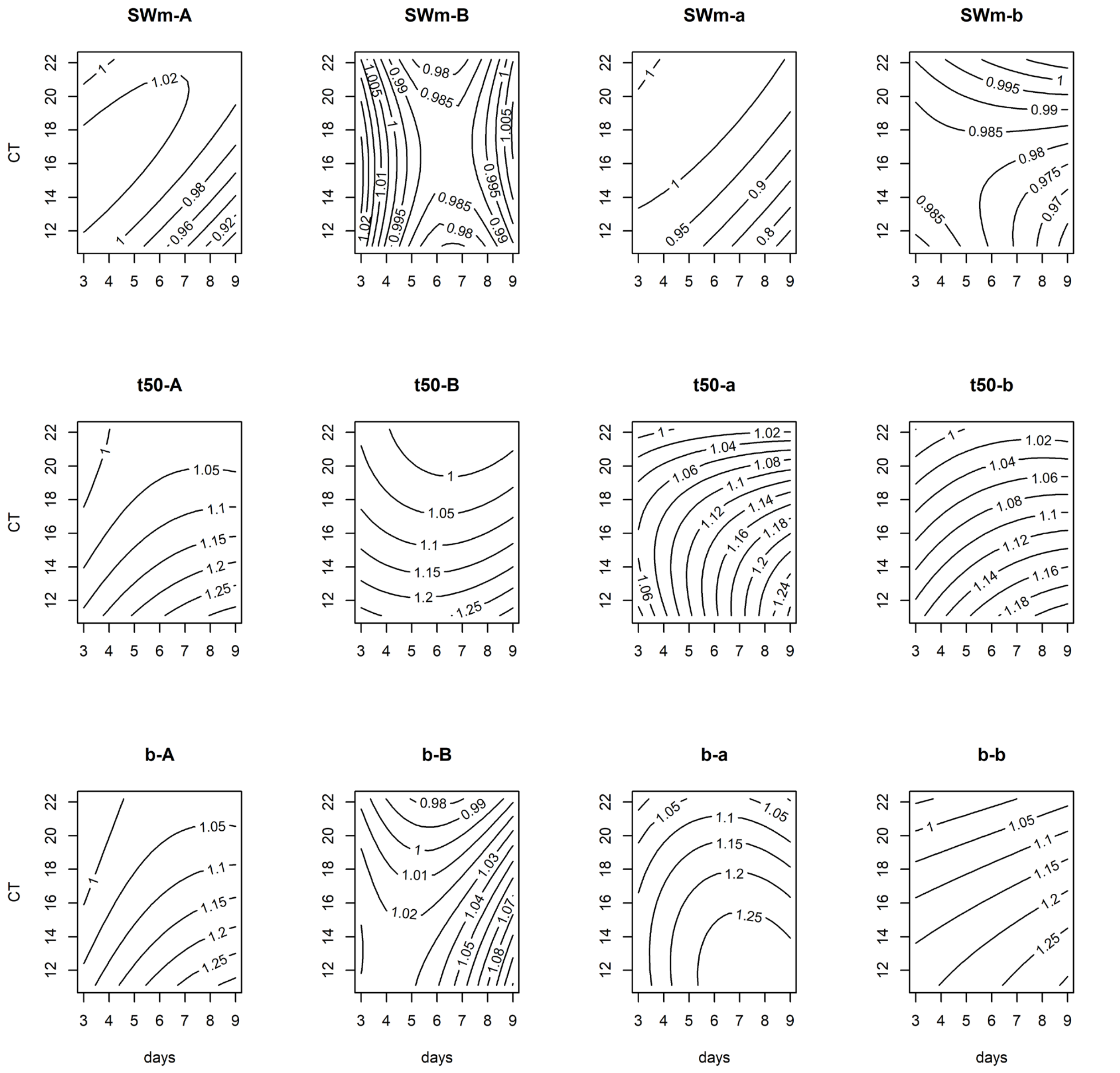

Figure S1. Contour plots for relative change in spikelet weight at maturity (SW_m), days from flowering to 50% grain filling (t_{50}) and shape or steepness of sigmoid curve (b) under varied low temperature stresses at flowering (A/a) and grain filling (B/b) stages in Huaidao 5 (A/B) and Nanjing 46 (a/b).

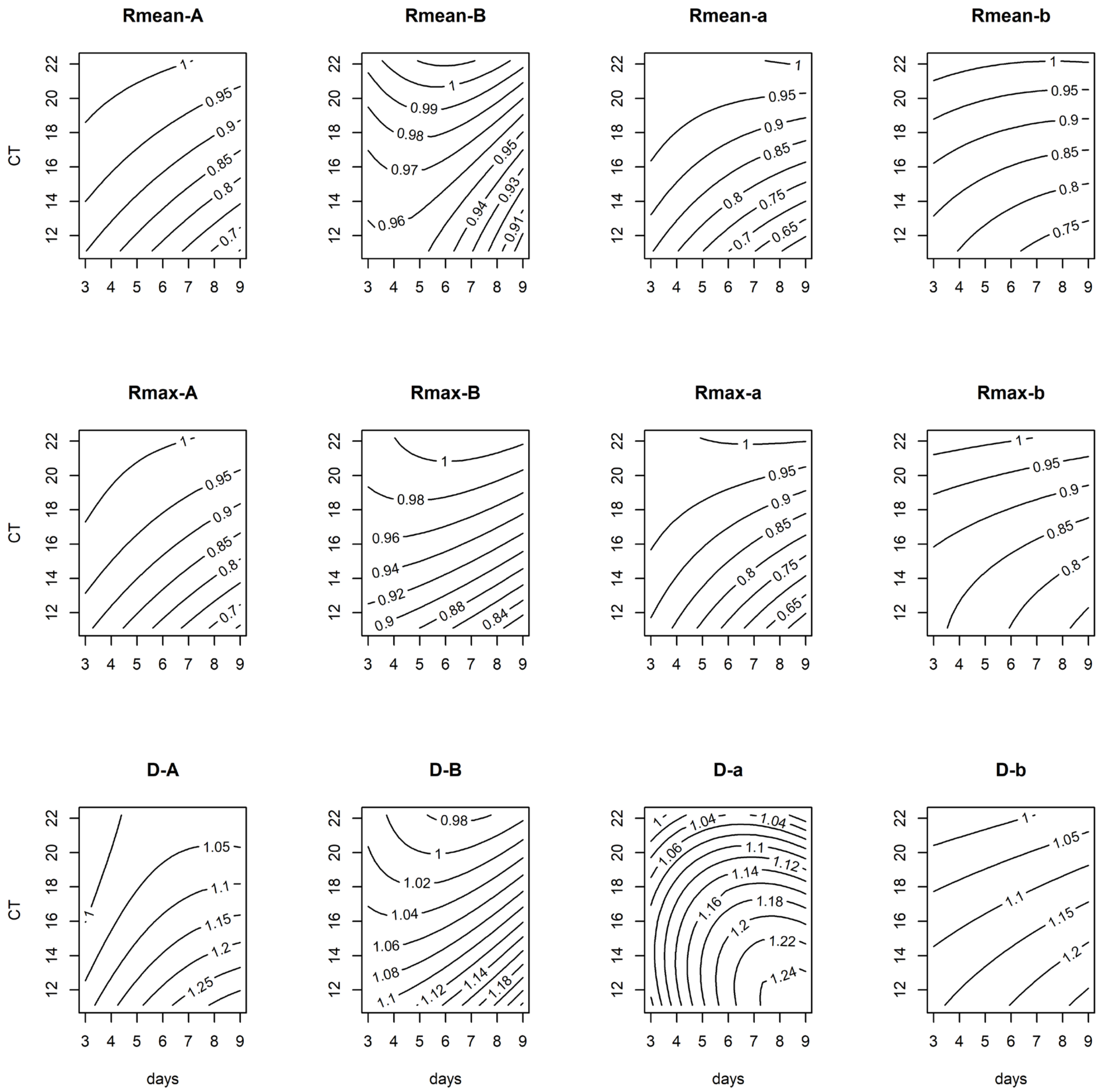

Figure S2. Contour plots for relative changes in maximum grain filling rate (R_{max}), mean grain filling rate (R_{mean}) and the total days from flowering to 95% SWm (D) under varied low temperature stresses at flowering (A/a) and grain filling (B/b) stages in Huaidao 5 (A/B) and Nanjing 46 (a/b).

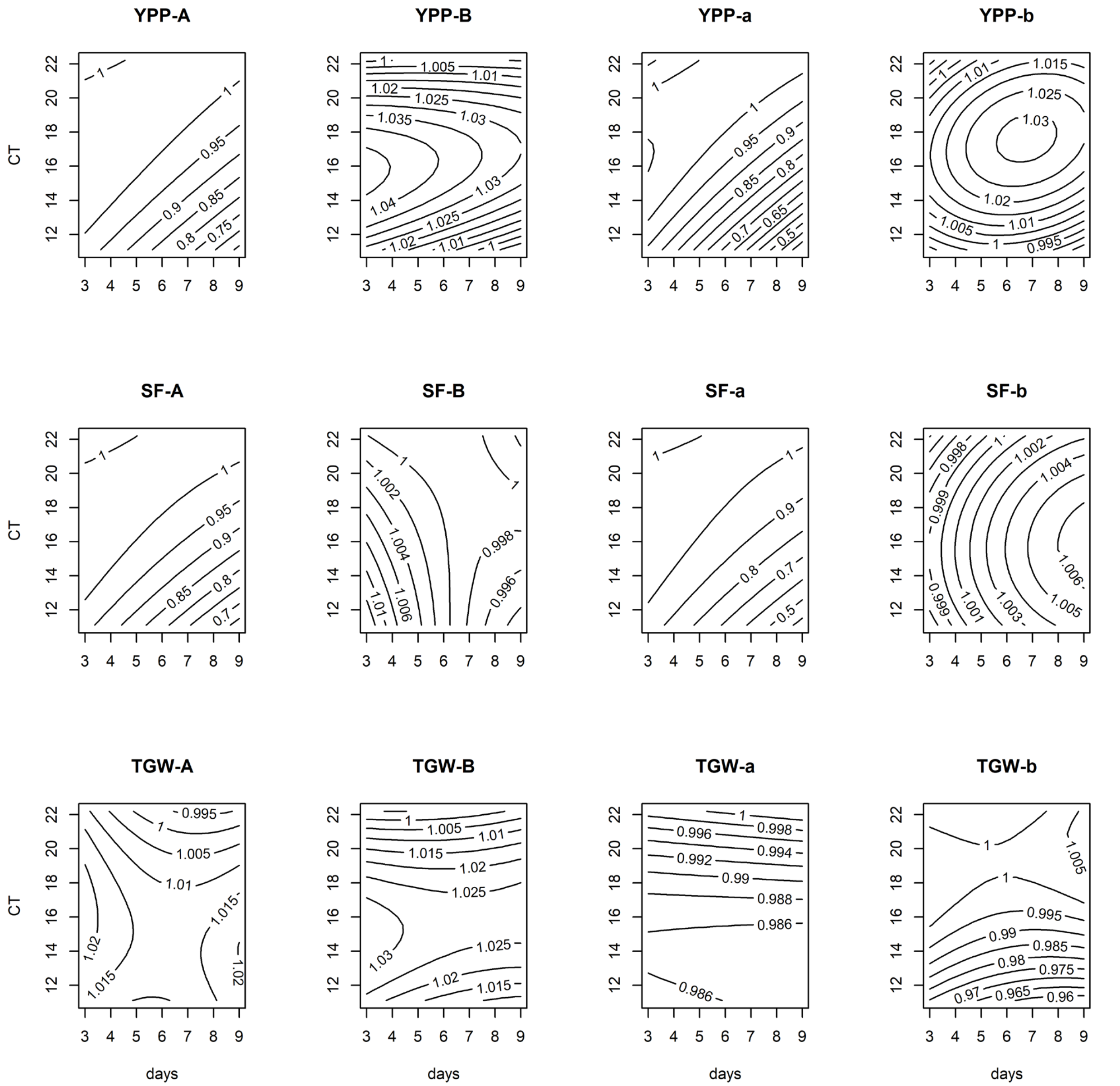

Figure S3. Contour plots for relative changes in yield per plant (YPP), spikelet fertility (SF) and thousand grain weight (TGW) under varied low temperature stresses at flowering (A/a) and grain filling (B/b) stages in Huaidao 5 (A/B) and Nanjing 46 (a/b).

Table S1. Variance analysis of grain yield and related parameters under post-heading low-temperature stress during two-year experiments in 2018-2019.

Source	SF (%)	SNPP	TGW (g)	YPP (g plant^{-1})
Year (Y)	1.12	11.51*	3.75	4.12
Cultivar (C)	175.32**	326.61**	970.2**	168.2**
Stage (S)	731.81**	2.66	15.27**	233.76**
Temperature (T)	199.75**	3.39	1.41	72.98**
Duration (D)	253.81**	0.01	0.27	102.43**
Y × C	0.13	0.44	0.01	0.26
Y × S	0.16	0.14	1.24	0.06
Y × T	1.91	2.39	0.99	1.62
Y × D	0.85	0.34	0.07	0.14
C × S	76.49**	0.1	10.7**	7.6**
C × T	13.94**	0.4	1.77	10.31**
C × D	12.79*	1.82	0.45	3.36
S × T	207.18**	5.79*	3.04	81.25**
S × D	255.95**	0.42	0.71	92.85**
T × D	71.07**	1.98	2.06	32.73**
Y × C × S	0.63	0.06	0.81	0.06
Y × C × T	2.04	0.29	0.08	1.54
Y × C × D	1.14	0.36	2.01	0.13
Y × S × T	2.23	1.2	3.12	4.17*
Y × S × D	1.51	0.36	1.54	0.17
Y × T × D	2.33	1.07	0.9	2.45
C × S × T	11.96*	0.73	0.75	4.46*
C × S × D	22.6**	0.54	0.56	6.73*
C × T × D	3.7	0.96	1.05	1.32
S × T × D	65.07**	1.84	1.61	15.36**
Y × C × S × T	2.19	1.38	0.56	1.57
Y × C × S × D	0.17	1.59	2.12	0.1
Y × C × T × D	2.43	1.19	1.18	0.84
Y × S × T × D	0.95	1.85	0.29	1.46
C × S × T × D	5.49*	0.75	0.54	3.79
Y × C × S × T × D	1.7	0.98	0.25	0.98

Note: The numbers in the table indicate the F values. * and ** indicate significant difference at $p<0.05$ and $p<0.001$, respectively. SF: spikelet fertility; TGW: thousand-grain weight; SNPP: spikelet number per panicle; YPP: yield per plant.

Table S2. Parameters of the response surface model fitted on yield as well as yield and grain filling related parameters under varied low temperature stresses

		**Huaidao 5**						**Nanjing 46**					
		**Flowering**			**Grain filling**			**Flowering**			**Grain filling**		
**Parameters**		**Estimate**	**SE**	**P value**	**Estimate**	**SE**	**P value**	**Estimate**	**SE**	**P value**	**Estimate**	**SE**	**P value**
SW_m	a	0.9074	0.2109	0.0051	1.0605	0.1437	0.0003	0.775	0.1771	0.0047	1.0908	0.0883	0
	b	0.0209	0.0231	0.4002	0.0101	0.0157	0.5447	0.0337	0.0194	0.1323	-0.011	0.0097	0.298
	c	-0.0238	0.0299	0.4562	-0.05	0.0203	0.0494	-0.0358	0.0251	0.2032	-0.0103	0.0125	0.4423
	d	0.0024	0.0009	0.039	0.0003	0.0006	0.6248	0.0041	0.0008	0.0016	0.0007	0.0004	0.1093
	e	-0.0009	0.0007	0.2137	-0.0004	0.0005	0.4583	-0.0014	0.0006	0.0508	0.0003	0.0003	0.3837
	f	-0.002	0.0021	0.379	0.0035	0.0015	0.0536	-0.0044	0.0018	0.0507	-0.0002	0.0009	0.8282
	R^2	0.7695	0	0	0.5663	0	0	0.9582	0	0	0.6226	0	0
t_{50}	a	1.2576	0.1822	0.0005	1.869	0.4019	0.0035	0.4616	0.2236	0.0845	1.097	0.1792	0.0009
	b	-0.0431	0.0199	0.0739	-0.0574	0.044	0.2393	0.0564	0.0245	0.0605	-0.0062	0.0196	0.7613
	c	0.1019	0.0258	0.0075	-0.0377	0.0569	0.5318	0.0812	0.0316	0.0425	0.0476	0.0254	0.1097
	d	-0.0028	0.0008	0.0108	-0.0018	0.0017	0.3289	-0.0031	0.001	0.0178	-0.0011	0.0008	0.1879
	e	0.0012	0.0006	0.0826	0.0013	0.0013	0.3506	-0.0015	0.0007	0.0729	-0.0001	0.0006	0.9119
	f	-0.0029	0.0019	0.1636	0.0056	0.0041	0.2201	-0.0007	0.0023	0.7659	-0.0015	0.0018	0.4424
	R^2	0.9661	0	0	0.8703	0	0	0.9312	0	0	0.9313	0	0
b	a	1.1	0.2855	0.0084	0.9902	0.1564	0.0007	0.4228	0.6447	0.5363	1.1159	0.6406	0.1321
	b	-0.0305	0.0312	0.3661	0.0115	0.0171	0.5265	0.0535	0.0705	0.4772	0.0128	0.0701	0.8611
	c	0.109	0.0404	0.0357	-0.0142	0.0221	0.5457	0.1547	0.0912	0.1409	0.0197	0.0907	0.8351
	d	-0.0028	0.0012	0.0626	-0.0012	0.0007	0.1181	-0.0023	0.0028	0.4298	-0.0004	0.0027	0.8764
	e	0.0009	0.0009	0.3729	-0.0003	0.0005	0.5711	-0.0018	0.002	0.4229	-0.001	0.002	0.6384

	f	-0.0031	0.0029	0.3211	0.0034	0.0016	0.0738	-0.0084	0.0066	0.2495	0.0005	0.0065	0.9425
	R^2	0.9222	0	0	0.8219	0	0	0.6887	0	0	0.7153	0	0
R_{max}	a	0.7988	0.1894	0.0056	0.9894	0.0898	0	0.7842	0.2939	0.0371	0.891	0.4127	0.0742
	b	0.0275	0.0207	0.2329	-0.0074	0.0098	0.4811	0.0325	0.0322	0.3507	-0.0045	0.0451	0.924
	c	-0.0684	0.0268	0.0435	0.0025	0.0127	0.8494	-0.1085	0.0416	0.0402	-0.0478	0.0584	0.4448
	d	0.0033	0.0008	0.0062	0.001	0.0004	0.042	0.0048	0.0013	0.0089	0.0013	0.0018	0.4979
	e	-0.0008	0.0006	0.2206	0.0002	0.0003	0.4492	-0.001	0.0009	0.3027	0.0006	0.0013	0.6742
	f	-0.0009	0.0019	0.6535	-0.002	0.0009	0.068	0.0005	0.003	0.8806	0.0013	0.0042	0.7708
	R^2	0.9675	0	0	0.9208	0	0	0.9491	0	0	0.8348	0	0
R_{mean}	a	0.7818	0.1208	0.0006	0.7407	0.1332	0.0014	0.9323	0.1479	0.0007	1.0529	0.2491	0.0055
	b	0.0346	0.0132	0.0397	0.0214	0.0146	0.1917	0.0163	0.0162	0.3529	-0.0231	0.0273	0.4295
	c	-0.0789	0.0171	0.0036	-0.0176	0.0188	0.3875	-0.1006	0.0209	0.003	-0.0348	0.0353	0.3612
	d	0.0037	0.0005	0.0004	0.0017	0.0006	0.0239	0.0053	0.0006	0.0002	0.0013	0.0011	0.2664
	e	-0.0011	0.0004	0.0272	-0.0006	0.0004	0.234	-0.0007	0.0005	0.1769	0.001	0.0008	0.2431
	f	-0.0006	0.0012	0.6371	-0.0015	0.0014	0.3117	-0.0011	0.0015	0.4783	0	0.0025	0.985
	R^2	0.9869	0	0	0.9484	0	0	0.9864	0	0	0.9167	0	0
D	a	1.1215	0.2245	0.0025	1.2327	0.2271	0.0016	0.4216	0.3695	0.2973	1.1097	0.3957	0.031
	b	-0.0344	0.0246	0.2107	-0.0163	0.0248	0.5354	0.0577	0.0404	0.2034	0.0038	0.0433	0.9338
	c	0.1156	0.0318	0.0109	0.014	0.0321	0.6779	0.1191	0.0523	0.0631	0.0275	0.056	0.6407
	d	-0.0032	0.001	0.0162	-0.0025	0.001	0.0436	-0.0023	0.0016	0.1938	-0.001	0.0017	0.5882
	e	0.001	0.0007	0.1983	0.0005	0.0007	0.525	-0.0018	0.0012	0.1782	-0.0005	0.0013	0.6977
	f	-0.0031	0.0023	0.2176	0.0031	0.0023	0.2237	-0.0053	0.0038	0.2067	0.0003	0.004	0.936

	R^2	0.9543	0	0	0.9041	0	0	0.8356	0	0	0.8179	0	0
YPP	a	0.6759	0.2095	0.018	0.795	0.024	0	0.5375	0.4947	0.3189	0.7619	0.2059	0.0101
	b	0.0632	0.0229	0.0329	0.0343	0.0026	0	0.0928	0.0541	0.1369	0.0273	0.0225	0.2714
	c	-0.09	0.0297	0.0232	-0.0094	0.0034	0.033	-0.155	0.0702	0.0694	0.0086	0.0292	0.7781
	d	0.0057	0.0009	0.0007	0.0005	0.0001	0.0028	0.01	0.0021	0.0033	0.0005	0.0009	0.6131
	e	-0.0024	0.0007	0.0106	-0.0011	0.0001	0	-0.0037	0.0016	0.0562	-0.0009	0.0006	0.233
	f	-0.0025	0.0021	0.2868	-0.0001	0.0002	0.591	-0.0044	0.0051	0.4127	-0.0013	0.0021	0.5738
	R^2	0.965	0	0	0.981	0	0	0.9364	0	0	0.3203	0	0
SF	a	0.7058	0.2625	0.0361	1.0542	0.0381	0	0.5525	0.4874	0.3002	0.9704	0.0406	0
	b	0.0597	0.0287	0.0825	-0.0019	0.0042	0.6579	0.0964	0.0533	0.1203	0.0028	0.0044	0.5538
	c	-0.0978	0.0373	0.0393	-0.0106	0.0054	0.0981	-0.1678	0.0692	0.0515	0.0027	0.0058	0.6501
	d	0.006	0.0011	0.0018	0.0003	0.0002	0.0793	0.0098	0.0021	0.0033	0	0.0002	0.9542
	e	-0.0023	0.0008	0.0303	0	0.0001	0.9399	-0.0038	0.0015	0.0493	-0.0001	0.0001	0.5036
	f	-0.0021	0.0027	0.4665	0.0003	0.0004	0.5025	-0.0033	0.005	0.5279	-0.0001	0.0004	0.7488
	R^2	0.9471	0	0	0.6531	0	0	0.9386	0	0	0.3938	0	0
TGW	a	0.9542	0.1075	0.0001	0.8978	0.1365	0.0006	1.0268	0.0635	0	0.8488	0.0527	0
	b	0.0107	0.0118	0.3984	0.0206	0.0149	0.2158	-0.0056	0.0069	0.4521	0.0198	0.0058	0.0137
	c	-0.0059	0.0153	0.7111	-0.0093	0.0194	0.6493	-0.0013	0.009	0.8858	-0.0141	0.0075	0.1084
	d	-0.0003	0.0005	0.5362	0.0003	0.0006	0.6101	0.0001	0.0003	0.77	0.0004	0.0002	0.1214
	e	-0.0003	0.0003	0.4056	-0.0007	0.0004	0.1443	0.0002	0.0002	0.376	-0.0006	0.0002	0.0137
	f	0.0008	0.0011	0.4765	0.0003	0.0014	0.8485	0	0.0006	1	0.0006	0.0005	0.3418
	R^2	0.3559	0	0	0.4369	0	0	0.4561	0	0	0.8885	0	0

YPP: yield per plant; SF: spikelet fertility; TGW: thousand grain weight; SNPP: spikelet number per panicle; SWm: spikelet weight at maturity; b: the shape or steepness of the sigmoid curve; t_{50}: days from flowering to 50% grain filling; D: days from flowering to 95% SW_m; R_{mean}: mean grain filling rate; R_{max}: maximum grain filling rate.
